# Ethics, law, and politics in palaeontological research: The case of Myanmar amber

**DOI:** 10.1038/s42003-022-03847-2

**Published:** 2022-09-29

**Authors:** Emma M. Dunne, Nussaïbah B. Raja, Paul P. Stewens, Zin-Maung-Maung-Thein, Khin Zaw

**Affiliations:** 1 GeoZentrum Nordbayern, Department of Geography and Geosciences, Friedrich Alexander University Erlangen-Nürnberg, Erlangen, 91054 Germany; 2 School of Geography, Earth & Environmental Sciences, University of Birmingham, Edgbaston, Birmingham, B15 2TT United Kingdom; 3 Department of International Law, Geneva Graduate Institute, Chemin Eugène-Rigot 2A, Geneva, CH-1211 Switzerland; 4 Department of Geology, University of Mandalay, Mahaaungmyae, 05032 Mandalay, Myanmar; 5 CODES Centre of Ore Deposit and Earth Sciences, School of Natural Sciences, University of Tasmania, Geology Building, Clark Rd, Private Bag 79, Hobart, TAS 7001 Australia

**Keywords:** Palaeontology, Ethics

## Abstract

Fossil material in amber from Myanmar can provide important insights into mid-Cretaceous forest ecosystems. However, Myanmar amber has been receiving increased international attention due to reported links between amber mining and the ongoing humanitarian crisis in northern Myanmar, as well as the legal issues associated with its exportation. Here, we conduct a bibliometric analysis of Myanmar amber publications (1990–2021) and demonstrate how research interest in Myanmar amber is explicitly linked to major political, legal, and economic changes. An analysis of the authorship networks for publications on amber inclusions reveals how current research practices have excluded Myanmar researchers from the field. In addition, the international trade of Myanmar amber with fossil inclusions falls into a legal ‘grey-zone’ which continues to be exploited. This case study vividly demonstrates that systemic changes, alongside an increased awareness of inequitable research practices amongst the broader scientific and allied communities, are urgently needed to curb illegal practices in palaeontology.

## Abstract (web summary)

Myanmar amber collection and study in palaeontology is clouded with ethical and legal issues. Here, the history and legality of Myanmar amber use in the literature is reviewed.

## Background

Fossils entombed in amber provide exceptional and unique insights into the morphology and evolution of organisms that inhabited past forest ecosystems^1^. Amber, which is fossilised plant resin, of various geological ages can be found in several parts of the world, including the Baltic region of Europe, Alaska, Madagascar, Lebanon, the Dominican Republic, and Canada. Amber from Myanmar (formerly Burma) is particularly famous for preserving the remains of insects, plants, and reptiles that lived alongside the dinosaurs during the mid-Cretaceous, approximately 99 million years ago^2^. However, Myanmar amber has more recently gained international attention due to legal and ethical issues in the discovery, collection, trade, and research of this material^3–5^.

**Fig. 1:**
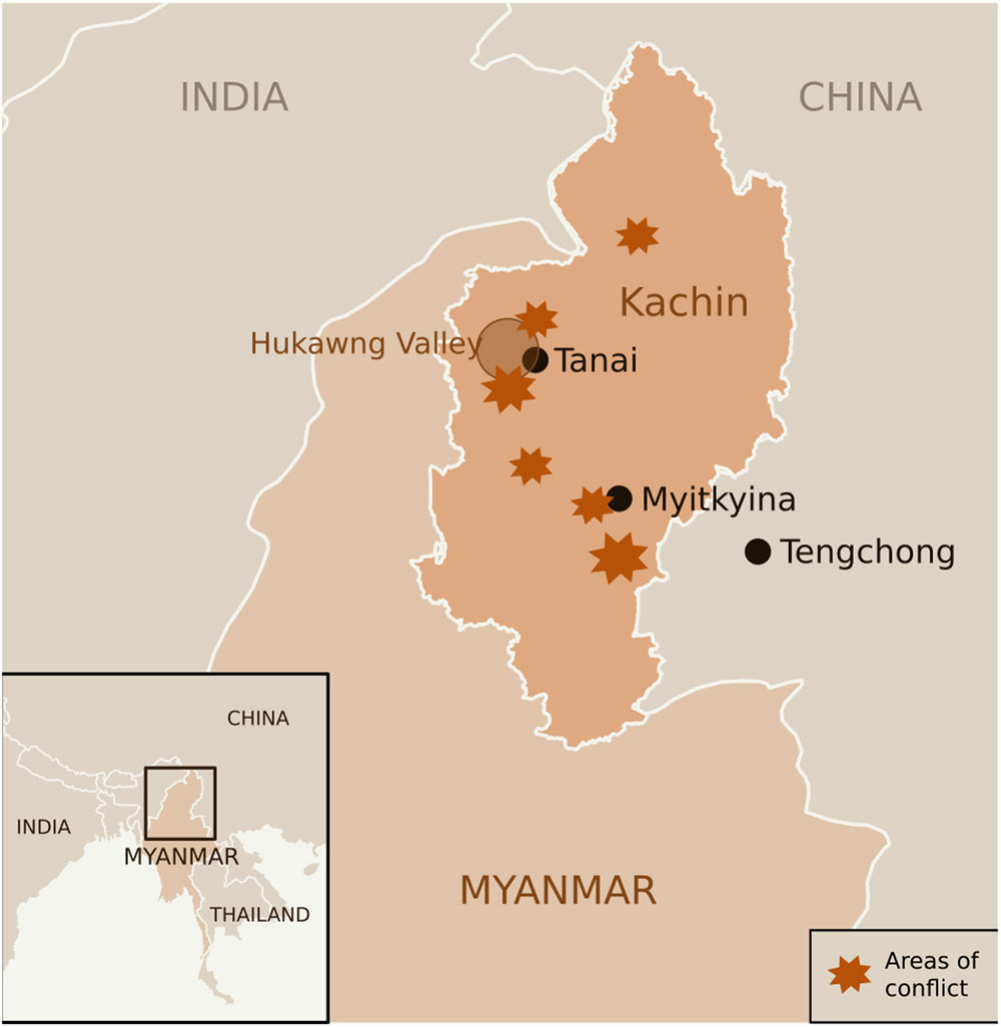
Locations in Myanmar associated with Myanmar amber and areas of major conflict. Location of Myanmar in Southeast Asia (inset) and location of Kachin state in Northern Myanmar (main). Many amber mines are located in Hukawng Valley (circled), close to the city of Tanai. Also included are the major amber trading cities of Myitkyina in Myanmar and Tengchong in Yunnan, China. Star icons denote major areas where conflict (e.g. armed clashes and violence against civilians) has been reported in July 2020 to December 2021 (sourced from The Armed Conflict Location & Event Data Project; www.acleddata.com).

Myanmar has a rich palaeontological heritage, including fossils of the oldest representatives of anthropoid primates^6^. But by far the most popular fossil finds from the country are those in amber. Most amber is mined in the northern state of Kachin, with many fossils coming from the Hukawng Valley (Fig. 1), though amber is also mined from several other regions^7,8^. To the international palaeontological community, this region is highly productive in terms of amber fossils, but on the ground, it has endured armed conflict between the Kachin Independence Army (KIA) and the Myanmar government military forces, the Tatmadaw, since the 1960s, only two decades after the country gained independence from British rule in 1948. Both the KIA and the Myanmar military have funded their activities through profits from the mining industry for decades, through both legal and illegal routes^9^. The most recent attempt by the military to seize control of the amber mining areas in Kachin State from the KIA occurred in 2017, and soon after, a UN Human Rights Council fact-finding mission reported on the violations of international human rights law and international humanitarian law perpetrated by the military in these areas^10^. These events have led recent media reports to describe working on Myanmar amber as an ‘ethical minefield’, and to question the ‘human cost’ of working on this material^3,4,11^.

Several countries have legislation in place to protect fossils from being exported illegally or studied without the involvement of local researchers^12^. More generally, this kind of legislation protects against extractive research practices, often referred to collectively as ‘parachute science’ (or scientific colonialism), whereby researchers from higher income countries collect specimens or data from lower income countries without engaging with local researchers or communities^13–15^. While Myanmar has national laws and is a signatory of several international conventions that could apply to fossil material in amber (Table 1, Box 1), the domestic regulation of this material’s export has a major loophole^16^. Most of the amber mined in Kachin State is sent across the Chinese border to Tengchong, where it is sold in large markets to jewellers, private collectors, and palaeontologists^4^. Permanent export of fossil material from Myanmar is prohibited under the 2015 Protection and Preservation of Antique Objects Law. The 1970 UNESCO Convention on the Means of Prohibiting and Preventing the Illicit Import, Export and Transfer of Ownership of Cultural Property^17^, an international treaty that entered into force for Myanmar in 2013, also protects cultural property from illicit trafficking (Table 1). For this purpose, the Convention obligates states parties to pass and enforce domestic legislation. However, under the 2019 Myanmar Gemstone Law (and its previous versions) amber is classified as a gemstone and its export can be legal if accompanied with the appropriate paperwork (Table 1). Neither the antiquities law (2015 The Protection and Preservation of Antique Objects Law; Table 1) nor the gemstone law (2019 Myanmar Gem Law; Table 1) explicitly mention amber with fossil inclusions, leading to it falling within a legal grey zone, where the gemstone can in principle be legally exported (with accompanying documentation), but not its fossilised contents.

**Table 1:** National laws and international treaties that apply to fossil specimens in amber uncovered in Myanmar.

Laws and treaties		Description
National legislation on gemstones	2019 Myanmar Gem Law	Builds on previous iterations of this law allows for larger scale extraction of gemstones, subject to approval of a licence by the government. Also specifically mentions “extraction royalties” that licensees must pay and the right to sell gems at government-approved gem markets.
	1995 Myanmar Gemstone Law (not in force)	Classifies amber as a gemstone and outlines the permits and permissions required to excavate and sell gemstones (including amber) both nationally and internationally; repealed by the 2019 Law.
National legislation on antiquities, including fossils	2015 The Protection and Preservation of Antique Objects Law	Defines antique objects as “tangible and intangible cultural heritage including fossil, corpse and bones of human beings and various types of animals”. Permission to carry out excavations must be sought from the Department of Archaeology and National Museum. Transport of antiquities to foreign countries without permission can result in imprisonment and/or fines.
	1957 Antiquities Act (not in force)	States that antiquities (which includes “any fossil remains of man or of animal”) are not to be excavated without permission from the then Director of the Burma Archaeological Survey. Any antiquities that are discovered must be immediately reported to the director, and export of antiquities is not permissible without prior permission; repealed by the 2015 Law.
International treaties	1954 Hague Convention for the Protection of Cultural Property in the Event of Armed Conflict with Regulations for the Execution of the Convention	Entered into force for Myanmar in 1956, its article 4(3) requires contracting states “to prohibit, prevent and, if necessary, put a stop to any form of theft, pillage or misappropriation of […] cultural property” in the event of an armed conflict.
	1970 UNESCO Convention on the Means of Prohibiting and Preventing the Illicit Import, Export and Transfer of Ownership of Cultural Property	Came into force for Myanmar in 2013. This treaty “urges States Parties to take measures to prohibit and prevent the illicit trafficking of cultural property. It provides a common framework for the States Parties on the measures to be taken to prohibit and prevent the import, export and transfer of cultural property.”
	1995 UNIDROIT Convention on Stolen or Illegally Exported Cultural Objects	In force in Myanmar since 2018. This treaty supplements the 1970 UNESCO Convention in regulating the private law implications of the restitution of cultural objects that had been stolen or illegally exported. The agreement inter alia provides that stolen cultural objects must be returned and that good faith purchasers are entitled to compensation. Besides, any state party may request another to return illegally exported cultural objects

For national laws, the legislation that is currently in force as well as the first legislation that came into force is listed. These and other amendments can be accessed through Myanmar Law Library (myanmar-law-library.org).

Due to this seemingly complex legal and ethical situation, the palaeontological community has not yet united on a stance towards the issues surrounding Myanmar amber. In 2020, the Society of Vertebrate Paleontology (SVP) released a letter to editors of palaeontological journal editors, calling for a moratorium on publishing material in Myanmar amber, particularly that acquired after the military took control of the mines in 2017^18^. Responses to these calls have been varied, with some journals declaring support for this move in the form of changes to their editorial policies^19,20^, while other palaeontologists have strongly disagreed^21,22^. In 2021, SVP released a second letter calling for a hard moratorium on the publication of fossil material in Myanmar amber obtained after the military coup earlier that year, as well as providing guidelines on researching amber material acquired before this date^23^.

Numerous publications on spectacularly preserved fossils in Myanmar amber enter the scientific literature each year, and the vast majority of these do not consider the ethical implications of studying this material^14^, in particular the links between amber mining in Kachin State and the documented human rights abuses by the military. In this study, we use bibliometric and affiliation data from the Web of Science to investigate temporal and geographic trends in Myanmar amber research over the last three decades (1990–2021) and explore how research output is related to national and international legal, commercial, and political developments. Through this investigation, we highlight the need for stronger systemic consideration of the ethical and legal concerns of working on this material, and palaeontological material from other conflict zones around the world.

Box 1 Legal Documents

- *International Treaties*
  ◦ Convention for the Protection of Cultural Property in the Event of Armed Conflict (adopted 14 May 1954, entered into force 7 August 1956), 249 UNTS 215.
  ◦ Convention on the Means of Prohibiting and Preventing the Illicit Import, Export and Transfer of Ownership of Cultural Property (adopted 14 November 1970, entered into force 24 April 1972), 823 UNTS 231.
  ◦ UNIDROIT Convention on Stolen or Illegally Exported Cultural Objects (adopted 24 June 1995, entered into force 1 July 1998), 2421 UNTS 457.
- *Domestic Legislation*
  ◦ The Myanmar Gemstone Law 1995 (The State Law and Order Restoration Council Law No. 8/95)
  ◦ The Myanmar Gemstone Law 2019 (Pyidaungsu Hluttaw Law No. 4/2019)
  ◦ The Antiquities Act 1957
  ◦ The Protection and Preservation of Antique Objects Law 2015 (Pyidaungsu Hluttaw Law No. 43/2015)

### Trends in Myanmar amber research and collaboration

**Fig. 2:**
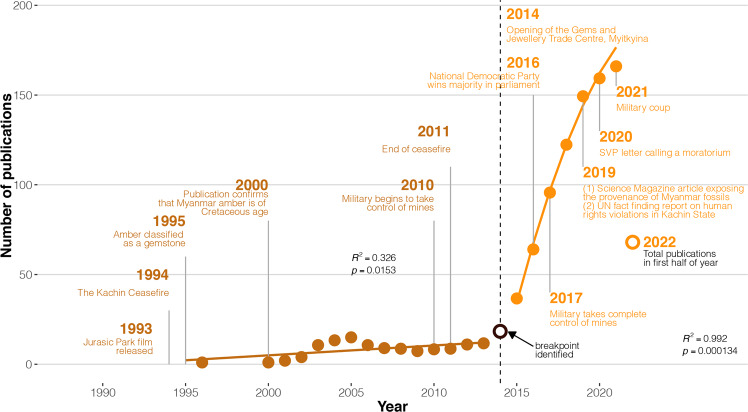
Trends in Myanmar amber publication activity including important political, legal, and commercial events since 1990. Temporal trends are based on a 3-year rolling average to allow time for the publication and peer review process.

Over the past three decades, the number of publications on fossil material in Myanmar amber recorded by Web of Science has increased (Fig. 2). A breakpoint is observed around 2014, dividing the time series into two independent periods: pre-2014 (1990–2013) where the increase in publications on Myanmar amber is slow but steady, and post-2014 (2014–2020) where there is a significantly more dramatic increase (Fig. 2 and Table S1). During the first period, pre-2014, the number of publications remained low, but still increased over time (*slope* = 0.55), with a peak observed around 2005. This changed after the year 2014 when the number of publications started rapidly increasing (*slope* = 24.8), reaching its highest value in 2020, with a total of 175 recorded publications in that year alone.

As a comparison, we examined the temporal trends in publications on non-amber fossils from Myanmar, such as Cenozoic primates and petrified wood^24,25^. We observed two breakpoints in the non-amber time series, at the years 2004 and 2016 (Fig. S1). The first publication on non-amber fossils appears in the year 1999 (according to Web of Science), and we see a slow increase over time (*slope* = 0.25) until 2004 when the number of publications starts to decrease at a similar rate (*slope* = −0.35) as the previous period (Fig. S1). Finally, we see an increase in the number of publications post-2016 (*slope* = 1.03). In general, the number of non-amber fossils publications remained quite low, especially when compared with counts of amber fossil publications over the same interval. Note that these identified breakpoints should be taken in the context of the low overall amount of data in this time series.

**Fig. 3:**
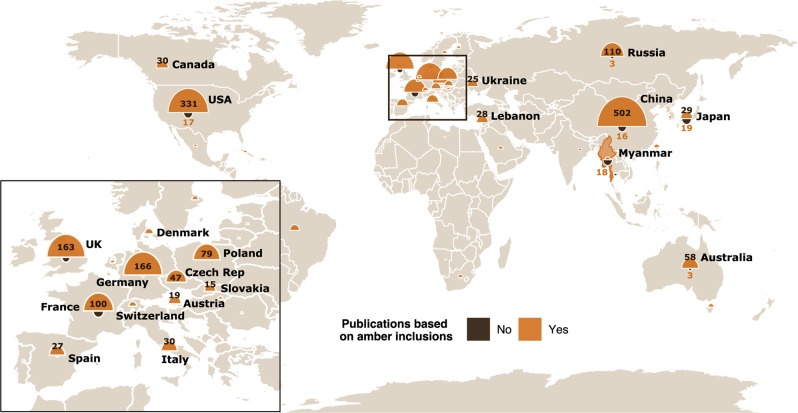
Countries where authors are based who have conducted research on Myanmar amber and non-amber fossils from Myanmar. World countries affiliated with authors who have conducted research on Myanmar amber (orange semi-circles) and non-amber fossils from Myanmar (brown semi-circles) from 2014–2021 when the number of publications on Myanmar amber was rapidly increasing year on year. An expanded view of the countries of Europe is shown in the inset.

Most of the research on fossils from Myanmar (~95%) is based on specimens in amber (Fig. 3), with China being a top contributor, followed by the US (Fig. 3). A considerable amount of research is also contributed by European-based authors, especially from Germany, the UK, France, and Poland. Only 3 out of 872 amber publications included co-authors from Myanmar. In contrast, for non-amber publications, the contributions of authors based in Japan (*n* = 18), Myanmar (*n* = 17) and the US (*n* = 16) are comparable. The number of amber publications post-2014 was 35 times higher than the number of non-amber publications, whereas pre-2014, they were only 5 times higher than non-amber ones. In general, the number of amber publications per country (based on author affiliations) was significantly higher (Wilcoxon test: *V* = 375, *p* < 0.05) than non-amber publications even after correcting for the expected difference between amber fossils and other fossils (standardised according to the period 2004–2015). No difference between amber and non-amber publications was observed before 2014 (Wilcoxon test: *V* = 191, *p-value* > 0.05).

**Fig. 4:**
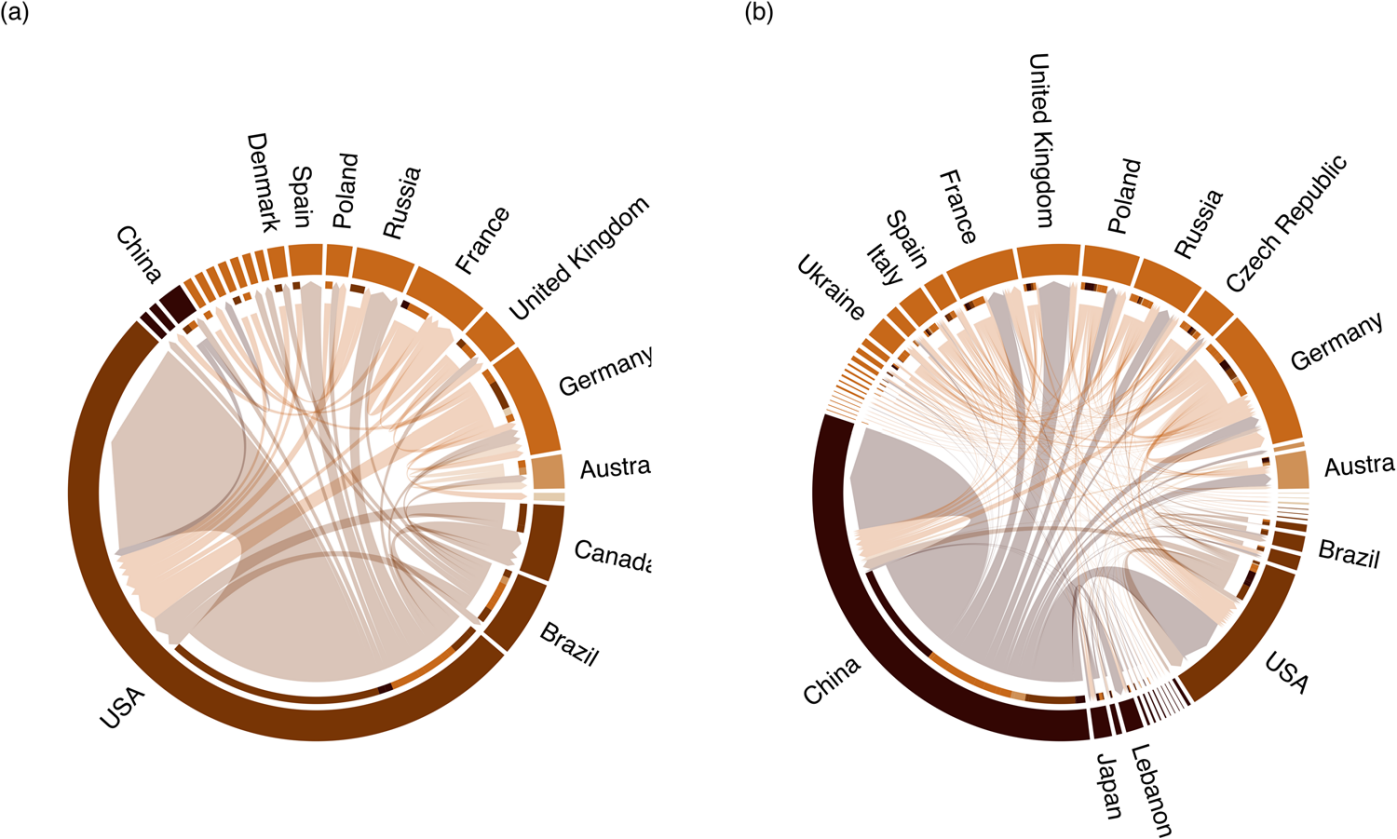
Collaboration networks between authors who publish on Myanmar amber both before and after 2014. Collaboration networks between countries (**a**) pre-2014 and (**b**) post-2014 (includes the year 2014). The chords represent connections between the affiliated country of the lead author (at chord base) and the affiliated countries of their co-authors (at the arrowhead). Chord thickness represents the relative number of publications co-authored by two particular countries where at least one country was the affiliate country of the lead author. Country’s segments and chords are coloured according to continental region.

Examining the network of collaborations between authors on amber and non-amber publications, we identified the countries with most power and influence over research trends. Until 2014, researchers from the US led in terms of first-authored amber publications (59%; *n* = 69) and held the most important position and influence when it came to collaborations, with mostly in-country collaborations occurring during that time (81%; Fig. 4). Since 2014, China has been the dominant country for Myanmar amber research (47%, *n* = 417) with the US moving to third position (9%, *n* = 79, Fig. 4 and S2), just behind Germany (9%, *n* = 81, Fig. 4 and S2). China was not in the top ten countries with lead authorship before 2014 (Fig S2). Multi-author amber publications were also more common post-2014, with only 10% (*n* = 93) of publications containing a single author after 2014, compared to 36% (*n* = 43) before 2014.

In-country collaborations (i.e. papers published by authors all affiliated with institutions in the same country) were more prevalent before 2014, with an average of 63% of collaborations being among researchers from the same country, compared with 46% after 2014. Other countries besides China and the US, such as Germany, Russia and Poland, also significantly contributed to amber research post-2014 (Fig. S3). However, when the collaboration network is analysed regardless of authorship order, Germany and the US have the highest betweenness and degree centrality measures both pre- and post-2014 suggesting that these two countries had a high degree of influence on information flow. China has the highest eigenvalue centrality post-2014, providing evidence for its overall influence in amber research (Fig S4).

### Political, legal, and economic factors drive research on Myanmar amber

The dramatic increase in Myanmar amber research output observed over the last two decades (Fig. 2) can be explicitly linked to major political, legal, and economic changes. Prior to the 2000s, only one publication on Myanmar amber was recorded in Web of Science: a paper from 1996, which described fossilised ant specimens housed at the Natural History Museum in London that were acquired during colonial times^26^. At the same time as this publication, the Kachin State mining business was undergoing a radical change. Prior to 1994, mines in Kachin State were under total control of the Kachin Independence Army (KIA). After a ceasefire agreement with the Tatmadaw (Myanmar armed forces), most mining areas in Kachin State were nationalised and placed under the control of the state-owned Myanmar Gem Enterprise, which regulated mining activity and issued mining licences^9,27^. In 1995, new legislation exclusively for gemstones, including amber, was introduced, allowing their extraction and marketisation (Table 1). In 1999, a Canadian mining company obtained amber pieces from former miners for radiometric dating purposes that were eventually sent to colleagues at the American Museum of Natural History (AMNH)^28,29^, kickstarting research on Myanmar amber and leading to the patterns that we observe today (Fig. 2). Myanmar amber, until this point was thought to be of Eocene age, but was ultimately confirmed to be mid-Cretaceous in age^30^. More amber was sold to the AMNH and some private collectors^27,31^, which resulted in several publications in the early 2000s (Fig. 2), mostly by researchers based in the US (Fig. 4). During this time, mining operations remained small-scale^27,28^.

In the late 2000s and early 2010s, trade with China began to gain momentum, and at the same time, Chinese amber mines tapped out^4^. Consequently, demand for Myanmar amber increased, leading to an expansion of mining operations in the Hukawng Valley^27,32^ (see Fig. 1). In 2011, fighting resumed between the KIA and the Tatmadaw—however, seemingly without affecting amber trade with China, which kept growing^27^. Reports seem to suggest that the mining and trade were controlled by a commonality of interest from the KIA and the Tatmadaw, where the KIA controlled the mines but the Tatmadaw regulated the different nodes of trade between Kachin State and China^27,32^. Both armed forces collected taxes on the mines and the transportation of amber and related materials (such as fuel, machinery, and food). As the fighting between the KIA and the Tatmadaw worsened in the lead up to the 2017 military offensive^33^, the KIA was forced away from the jade and gold mines that they controlled, meaning revenues from amber became even more important.

The year 2014 marked the beginning of a dramatic increase in Myanmar amber research output (Fig. 2). This increase correlates with the increased availability of Myanmar amber, particularly on the Chinese market. Much of the amber mined in Kachin State that is taken across the border to be sold in the markets of Tengchong, China (Fig. 1), is outside of Myanmar’s regulatory landscape, as the cost of custom tax was not deemed to be profitable for traders^27^. Once in Tengchong, amber can be legally sold on the Chinese market and is subject to state regulations there. Amber can also be bought from the city of Myitkyina in Myanmar (Fig. 1), especially at the Gems and Jewellery Trade Centre, which opened in 2014^34^. However, this tends to be a less lucrative option for Chinese dealers as amber prices tend to be higher here than across the border in Tengchong. As amber availability increased on the Chinese market, the material became more accessible to Chinese palaeontologists and private collectors. These factors combined likely led to the increase in Myanmar amber publications seen from 2014 onwards (Fig. 2). The commercial availability of Myanmar amber is also likely a large factor driving the difference in research interest between amber specimens and non-amber specimens (Fig. 3). In order to obtain non-amber fossils, fieldwork and//or access to fossil collections is necessary, and combined with political events, especially across recent years, this can be prohibitive for collaborations that require international travel.

Prior to 2014, US researchers dominated research output, whereas after 2014, Chinese researchers have a monopoly (Fig. 4). Both before and after 2014, many countries in Europe, such as the UK, Germany, France, and Poland, as well as Australia and Brazil, retained a share of the research output on Myanmar amber (Fig. 4). This is likely due to their legacy of expertise on the topic of both Myanmar amber and amber from other regions (e.g. Baltic and Dominican amber), as well as on the topic of palaeoentomology (study of fossil insects, which are frequently found preserved in amber). Several of these countries also dominate research output on non-amber fossils from Myanmar (Fig. 3), which is related to their general positions as centres of power and knowledge in the field of palaeontology more broadly^14^.

The total number of publications on Myanmar amber published in 2021 (*n* = 157) did not surpass the 2020 total (*n* = 175) (Fig. 2). The total for 2022 (*n* = 68 at the time of writing) also does not seem set to surpass the 2021 total. This might indicate the beginning of a change in publishing trends and a diminished interest in Myanmar amber research, perhaps due to increased scrutiny from the palaeontological community. This could also be a result of knock-on effects from the COVID-19 pandemic, which has impacted the scientific publishing process and restricted international travel, particularly travel to museums, other research institutions, and field sites.

### Palaeontological research and collaboration in Myanmar

While the post-2014 boom in amber trade increased the accessibility of fossil specimens to Chinese researchers, and by extension, to other international researchers, it failed to do so for local Myanmar palaeontologists (Figs. 3 and 4). The development of the research landscape in Myanmar goes hand in hand with the political changes that have occurred in the country since independence from the United Kingdom in 1948^35^. Until the 1962 coup that marked the beginning of the political dominance of the military in the country, post-colonial Myanmar experienced a boom in university admissions and the government was making various investments in the academic sector^36^. Following this coup, universities lost autonomy over their budgets, and tighter restrictions were put in place on research and travel by Myanmar scholars as well as foreign academics^35^. Such restricting and isolationist policies remained in place, curbing research and academic exchange among scholars, until political reforms in the late 2000s and early 2010s, in the lead up to 2015 democratic elections. This is reflected in the research output by Myanmar researchers, which remained low until the early 2010s (Fig. S5), and also in the research of non-amber fossils found in the country during that time (Fig. S1).

In Myanmar, international research collaborations are vital for strengthening palaeontological research, and scientific research more broadly, and so it is critical that these international partnerships are equitable and sustainable^5^. The Eocene-age Podaung Formation in Myanmar, which contains fossils of the oldest representatives of anthropoid primates (the taxonomic group that includes monkeys, apes, and humans), has been the focus of several field efforts in the last century. The first fossils were collected in 1914, but no further work occurred until the late 1990s^6,37^. Given the attention that such fossils from Myanmar and other Asian regions were getting at the time, the government organised a fieldwork expedition to the Podaung Formation in 1997, aiming to “enhance the stature of the country”^38^. In the following years, joint fieldwork expeditions took place between Myanmar research teams and teams from the US, Japan, and France. Many of these international collaborations persist to this day, resulting in several publications over the years (e.g^6,39^.). Critically, we found that many of these publications on primate (i.e. non-amber) fossils include researchers from Myanmar as co-authors, which is in stark contrast to amber publications that rarely indicate collaborations with local researchers (Fig. 3).

Only five out of 872 (0.06%) publications on Myanmar amber in our dataset include researchers based in Myanmar as co-authors (Fig. 3). For three of these co-authored publications, the authors listed as being affiliated with an institution in Myanmar have Chinese institutions as their primary affiliations. Their Myanmar affiliations are listed as the Southeast Asia Biodiversity Research Institute in Myanmar, which was established by the Chinese Academy of Sciences and the Myanmar Ministry of Natural Resources and Environmental Conservation in 2013 through bilateral agreements with the aim of carrying out collaborative research and providing training to young people in South East Asia^40^. Only two of these publications have authors with primary affiliations in Myanmar. The first lists an author based at the Myanmar Amber Museum in Yangon (myanmarambermuseum.org), an institution which also sells amber specimens. The other discusses the issue of parachute science with respect to Myanmar amber research, written by two authors of this study^5^. What we observed here is an extreme form of parachute science where instead of fieldwork, amber specimens are obtained through commercial routes and are apparently not regulated accordingly by national laws relating to fossils or gemstones (Table 1). As such, the mechanisms driving research on Myanmar amber are distinct from those driving other palaeontological work that conforms to national legislations and promotes collaboration between Myanmar and foreign researchers. Curbing parachute science and other unethical or illegal research practices is of the utmost importance as this leads to the erasure of invaluable local expertise and perpetuates the global power and knowledge imbalances in palaeontology^12,14^.

### Legal and ethical considerations for Myanmar amber research

The large amount of amber that is moved across the Chinese border to be sold to jewellers, collectors, and palaeontologists indicates that Myanmar’s enforcement of its national laws is seriously deficient^16^, suggesting that it insufficiently implements the UNESCO Convention which not only requires adopting export restrictions on cultural objects (Art. 6(b)) but also the imposition of penalties or administrative sanctions on individuals violating those restrictions (Art. 8). However, the current Myanmar amber economy also strongly relies on China who also is a party to the 1970 UNESCO Convention and the 1995 UNIDROIT Convention (see Table 1). As such, China has an international legal obligation to prevent illicit trafficking of cultural objects, which is likely being violated while tons of amber are smuggled into the country. If Myanmar, as a party to the UNESCO Convention whose cultural patrimony is in jeopardy, were to call upon China as a state party also affected by this situation, China would even face an obligation to cooperate to bring an end to this threat to Myanmar’s cultural patrimony (Art. 9 UNESCO Convention). Some countries, such as Brazil, Argentina, and Mongolia, have national laws that control the export and study of fossils. The presence of these kinds of laws in a country often correlates with more domestic production of palaeontological research (as opposed to the fossils being studied by researchers based outside the country of origin)^13,14^. This demonstrates the need for governments and law enforcement to work alongside research institutions, museums, palaeontologists and fossil collectors in order to ensure that existing national laws are adhered to and updated based on best practices from other countries.

Scientific publications describing fossil specimens in Myanmar amber rarely, if ever, provide evidence that their material was obtained legally. Since the letter released by the Society for Vertebrate Paleontology SVP in April 2020^18^, the legal issues associated with Myanmar amber have been even more prominent within the palaeontological community. Despite this, only 9 out of 222 (4%) publications released since this date (i.e June 2020–June 2022) refer in any way to legal or ethical issues associated with the material they describe (Table S3). Among these, only two^41,42^ provide a detailed description of how their specimen was ethically acquired and accessioned alongside details of documentation to support this. In both cases, this procedure is in line with guidelines set out by SVP, discussed in more detail below^18,23^. The majority of these statements appear to distance themselves from the legal and ethical issues associated with Myanmar amber by declaring the specimens were collected ethically and/or legally, but without providing any documentation or evidence of this (Table S3). Furthermore, the majority of the publications that we examined only provided vague information about the location of the fossil material, for example “obtained from a mine” or “collected from Hukawng Valley”, without further supporting material such as coordinates or a map, likely because this information was not recorded in the first instance during collection. The absence of this information can lead to the loss of important contextual data (e.g. ability to geologically date the material), making this research difficult to critically analyse or even reproduce.

Numerous recent media reports have described Myanmar amber as an ‘ethical minefield’ and note the ‘human cost’ of working on this material^3,4,11^. In the lead-up to the takeover of the mines by the Tatmadaw in 2017, several attacks were launched in the amber mining regions, displacing thousands of people, who lost their homes and livelihood^32^. While the government claimed that its actions were to protect the environment, the aim may have been to control the mining operations given the ever-increasing demand and popularity of amber^27^, especially when a dinosaur tail preserved in amber was found and published at the end of 2016 (Fig. 2). The United Nations (UN) condemned the military offensive, and a UN fact-finding mission reported violations of international human rights law and international humanitarian law in Kachin State, including forced labour, torture, abductions, and sexual violence^10^. Amber continues to be mined in hazardous and dangerous conditions, as a result of both the nature of the mine shafts and the continuing conflict in the region^43,44^. According to recent reports, illegal mining for amber (as well as for gold and jade) in Kachin State by groups affiliated with the military has intensified since the coup in early 2021, which has led the UN to suspend climate work with the Myanmar government^45^. As of August 2021, the state of emergency declared following this year’s military coup is set to remain in place until at least August 2023^46^.

Some authors have said that there is no evidence that the military generates any significant revenue directly from amber containing fossils^47^. Given that most of the trade in these specimens occurs within a shadow economy where revenues go largely undeclared^27^, it would indeed be difficult to estimate the true revenue that the military or other parties receive. However, it is indisputable that amber is a significant source of income for the military^27,32^, and that fossil finds have directly influenced the increase in amber trade^32^ (Fig. 2). During the military takeover of the mines in 2017, the Tatmadaw collected taxes on all amber transport routes into China–a route often taken by fossils in amber–where the annual value of the trade is estimated at 1 billion USD a year^32^. Later that year the Tatmadaw moved to take over the amber trade at the source, instigating a humanitarian crisis^10,32^. The palaeontological community must acknowledge the contribution of research interest in Myanmar amber to the larger scale issues occurring in the country.

### Ethical and equitable research in palaeontology

In February 2022, SVP released guidance documents on carrying out research and publishing on amber material for researchers, research institutions, and journals, their editors and peer reviewers (available at: https://vertpaleo.org/governance-documents/). These include specific guidelines on the considerations researchers should take when preparing and reviewing manuscripts, including ensuring that appropriate documentation is available to attest to the provenance of amber specimens. It is yet to be seen whether these guidelines will be adopted widely by researchers aiming to carry out research on amber or by journals catering for such research. In addition, SVP revised the 2020 moratorium on specimens acquired post-2017; instead, authors are now recommended to provide documentation for the legal and ethical acquisition of their amber specimens. At the same time, SVP also called for a moratorium for any amber material acquired after the military coup, which began in February 2021 and is still ongoing at the time of writing.

The most ethical and equitable research projects feature input from all involved parties, both local and foreign, in order to develop research agendas that are built on mutual respect and the needs and interests of local people^12,14,48^. Researchers who are working on amber specimens from Myanmar should, therefore, reach out to Myanmar-based researchers who can provide greater expertise on the geology and palaeontology of the area of interest. Due to the drain in material and the high cost (relative to funds held by researchers in Myanmar) of acquiring amber specimens, local researchers are excluded from working on these specimens themselves, despite them originating from their home country (Figs. 3 and 4). This inhibits the development of local expertise and exacerbates the systemic inequalities presented here and in palaeontology more widely^12,14,49^. Researchers based in Myanmar are contactable through the usual professional channels (e.g. institutional webpages, publication authorship details, and academic social platforms). In fact, the authorship for this paper was established through cold emailing, and the entirety of the communication for this research collaboration was conducted virtually.

Museums can also play a part in paving the way towards more ethical research on Myanmar amber. Many museums, particularly in the US and Europe, already house large collections of Myanmar amber specimens. Museums that are part of the International Council of Museums (ICOM) should adhere to the ICOM Code of Ethics for Museums^50^ that lists the basic principles for acquisition of any material and the need for due diligence. Raising awareness about and supporting the fight against illicit trafficking of cultural material is one of ICOM’s highest priorities and by extension, that of its members. Museums can also assist in the establishment of local repositories in Myanmar, facilitate access and sharing of material, and provide training to Myanmar-based researchers and students.

### Future outlook and broader implications for palaeontological research

The case of fossil material in Myanmar amber is a clear example of exploitative (or ‘extractive’) research in palaeontology, centred around the principles of ‘parachute science’ and scientific colonialism. Our findings document a direct link between research output and political, legal, and economic changes happening within the country. The situation surrounding Myanmar amber is complex and ever-evolving, but nonetheless shows how scientific research does not happen in a vacuum, and is regularly impacted by several interconnected external factors. For the field of palaeontology, this case highlights the importance of conducting and promoting ethical, equitable, and sustainable research practices that centre the needs and interests of local communities. Researchers must also take time to become familiar with the regulatory and cultural landscape of the location where they intend to conduct field research to avoid conduct that is unethical and/or illegal. International collaborations should always include local researchers, who can provide unparalleled expertise and insights, even beyond scientific knowledge^13,14^. Additionally, funders, societies, editors, and peer reviewers must remain vigilant about ethical and legal issues in palaeontology to avoid perpetuating exploitative research practices (‘parachute science’). Palaeontology, and the geosciences more broadly, are currently grappling with overdue conversations around the societal and economic impact of their research practices, and action is urgently required. No matter how tenable the link between the scientific work and reported violations, our understanding of ancient life and its evolution should never come at the expense of human rights and human lives.

## Methods

### Data

Bibliometric details of palaeontological publications on Myanmar were downloaded from the Web of Science on the 8th of June, 2022^51,52^. Publications on Myanmar amber (amber publications, *n* = 937, spanning the years 1996–2021) and Myanmar fossils that are not encased in amber (non-amber publications, *n* = 55, spanning the years 1999–2021) were queried separately using specific keywords and filtered for categories pertaining to palaeontology (Table S2). The publications for which affiliations data were missing were manually obtained from the publisher’s webpage or from archived version of the manuscript reposited on servers such as ResearchGate or Academia.edu. Only 3 publications, all amber related ones, still had missing affiliations.

### Temporal trends in publications

We calculated a 3-year rolling mean of the number of both amber and non-amber publications during the period 1990–2021 to take into account the publication process from acquiring the material to carrying out the analyses to publication in a peer-reviewed journal. The 2022 datapoint was omitted as it only represents the number of publications for half of the year of 2022. A segmented regression with breakpoints analysis was performed on the time series of mean number of publications to identify the years where the temporal patterns in publication significantly changed using the R package *segmented* v1.3.4^53^. The *segmented()* function identifies potential breakpoints and calculates one or more segmented relationships from a linear model. This is achieved through an iterative process that only requires the initial values for the potential breakpoint(s)^54^. The initial values were obtained based on a visual inspection of the time series as well as the Davies test^55^. The Davies test is an independent test which verifies whether a non-constant regression parameter exists in a linear prediction, i.e. whether there is a significant change in slope between *k* number of points. The Davies test was repeated on identified segmented regressions until no more breakpoints were statistically deemed to be present. The Davies test also returns the best estimate among the *k* points, which was then fed into the *segmented()* function which calculates the actual breakpoint using a maximum likelihood algorithm. After this analysis, the study period was divided into the identified segmented periods based on the detected breakpoints. These breakpoints were then compared to changes that occurred in the economic and political situation in Myanmar to identify probable causes for the shifts observed in the palaeontological research landscape.

### Amber vs non-amber publications

Because non-amber fossils are without any doubt subject to legislation with regards to collection and export, we consider non-amber publications to be the control group that represents how these materials would be researched if the special circumstances under which amber material fall under would not exist. Given the disproportionate amount of amber publications to non-amber publications (likely a results of the increased availability of amber specimens), we standardised the number of amber publications according to a ratio of amber vs non-amber publications calculated over a certain period of time, identified by the segmented regression analysis. We then compared the standardised number of amber publications to the number of non-amber publications using a Wilcoxon signed-rank test.

### Position and influence in amber research

We also investigated the influence that certain countries hold over others when it comes to collaborating on publications about Myanmar amber during the segmented periods identified by the breakpoint analysis. We consider lead authorship to be a representation of influence in this particular study as usually for any publication, the lead author (and by extension, their institution or affiliate country) has the most responsibility and power over the line of research within the scope of the publication. As such we looked only at collaborations between the first author and the co-authors on a publication to identify the nature of the relationship between the countries of these authors. A country (country A) is considered to be hold more influence over another country (country B) when the number of publications led by researchers from country A is greater than the number of publications led by country B. We also constructed an undirected collaborative network using all affiliate countries regardless of their position on the authorship list to identify important actors within the network. We use centrality measures such as betweenness centrality and degree centrality as indicators of power. We also looked at whether more in-country collaborations occurred than between countries. Finally, we examined if there were certain countries that stood out in terms of number of leading publications through outlier detection using box plots as per Tukey’s method^56^.

### Reporting summary

Further information on research design is available in the Nature Research Reporting Summary linked to this article.

## Supplementary information

Supplementary Information

Reporting Summary

## Data Availability

Bibliometric data for publications on Myanmar fossils are available from the Web of Science using keywords outlined in Table S2. All relevant bibliometric datasets supporting the analyses presented in this study are available here: 10.17605/OSF.IO/P28XS. Myanmar laws (in both Burmese and English) can be accessed through Myanmar Law Library (myanmar-law-library.org).

## Code availability

All relevant code and data supporting the analyses presented here are available on Open Science Framework through: 10.17605/OSF.IO/P28XS.
