# Supplementary material for: Ethics, law, and politics in palaeontological research: The case of Myanmar amber
Source: Commun Biol. 2022 Sep 29;5:1023. doi: 10.1038/s42003-022-03847-2 (PMC9522859; doi:10.1038/s42003-022-03847-2)
Supplement: Supplementary file 1 — Supplementary Information [file 42003_2022_3847_MOESM1_ESM.pdf]

Supplementary Information

for:

Ethics, law, and politics in palaeontological research: The case of Myanmar amber

Emma M. Dunne, Nussaïbah B. Raja, Paul Stewens, Zin Maung Maung Thein & Khin Zaw

---

This document contains:

Supplementary tables S1 – S3

Supplementary figures S1 – S5

Table S1. Results from Davies' test to identify a change in slope in a linear regression. The test is repeated on the generated segmented regression after a breakpoint is identified (when $p < 0.05$) until no breakpoints are deemed to be statistically present (when $p > 0.05$). The column "best at" represents the point in the variable at which the maximum test statistic occurs. The "actual breakpoint" is the breakpoint then identified using a log-likelihood function.

**Publication type**	**Davies test**			**Actual breakpoint**
	statistic	*p-value*	*best at*	
Amber	21.7	6.4E-13	2014.8	2013.9
	-2.92	0.09	2019	NA
Non-amber	6.42	9.71E-05	2016.9	2016.4
	-3.42	0.03	2004.2	2004.0
	1.83	0.40	2006.3	NA

Table S2. Query details for bibliometric data obtained from the Web of Science.

**Publication type**	**Keywords**	**Categories**	**Query Link**
Amber	'Myanmar amber' OR 'Kachin amber' OR 'Burmese amber'	Paleontology, Geology, Zoology, Entomology, Evolutionary Biology	https://www.webofscience.com/wos/woscc/summary/398c1e91-c98f-47fe-ad3c-bf37eab44aed-019a8e6d/relevance/1
Non-amber	'Myanmar AND paleontology' OR 'Myanmar AND palaeontology' OR 'Myanmar AND paleobiology' OR 'Myanmar AND palaeobiology' NOT 'amber'	Paleontology, Geology, Zoology, Entomology, Evolutionary Biology	https://www.webofscience.com/wos/woscc/summary/2a75fa0c-3edc-4937-9823-a530d1e3be22-0152a0ec/relevance/1

Table S3: Example ethics declarations used in publications since May 2020 (following the release of a letter by the Society of Vertebrate Paleontology)

Publication	Statement
Daza *et al.* (2020) *Science*	“Specimens were acquired following the ethical guidelines for the use of Burmese amber set forth by the Society for Vertebrate Paleontology (see “Provenance and Ethical Statement” section of the supplementary materials for a detailed description of ethical fossil acquisition and accession). We hope that this study will serve as a model for other researchers working with these types of materials in this region.” - extended in supplementary material document.
Badano *et al.* (2021) *Insects*	“We are aware of the ethical issues involving Burmese amber and we declare that the specimen was collected before the humanitarian crisis that started in the excavation areas in 2017. The specimen is deposited in the Nanjing Institute of Geology and Palaeontology (NIGP), Chinese Academy of Sciences at Nanjing, China, in full compliance with the recommendations of ICZN, and the instructions of the International Palaeoentomological Society. The material examined during the present study was borrowed from the NIGP with the assurance that it had been acquired and imported in compliance with all local procedures and regulations”
Luo *et al.* (2021) *Insects*	"To avoid any confusion and misunderstanding, all authors declare that the fossil reported in this study was not involved in armed conflict and ethnic strife in Myanmar. This specimen is deposited permanently in a publicly owned collection in a national museum, in full compliance with the International Code of Zoological Nomenclature and Statement of the International Palaeoentomological Society"
Čerňanský *et al.* (2022) *Scientific Reports*	"The specimen was acquired ethically from a government licensed gem dealer in 2019, in a Myanmar government approved show, and was subsequently exported legally from Myanmar. In this paper, we follow a very strict protocol as to the origin of the amber piece, its acquisition, and the legalization of its final repository. The material came from a non-conflict zone in the Hkamti area and, at the time of acquisition, the Sea Sun Star company dominated the mining operations. This company is not listed in the United Nations Human Rights Council report as being involved in the Myanmar conflict. Detailed information on the ethical acquisition of PMF Ref-29689 specimen can be found in the following link: https://bit.ly/2x8gnVj. Paper trail, invoices, and customs forms are also available from the Peretti Museum Foundation website (www.pmf.org)."
Luo *et al.* (2022) *Fossil Record*	"To avoid any confusion and misunderstanding, all authors declare that the fossil reported in this study was collected before 2016 and was not involved in armed conflict and ethnic strife in Myanmar. This specimen is deposited in a public collection permanently that is always accessible to scientists by contacting the corresponding author, in full compliance with the International Code of Zoological Nomenclature and the instructions of

	the International Palaeoentomological Society"
Magnussen *et al.* (2022) *Zoological Journal of the Linnean Society*	"There is an ongoing ethical debate on the usage of Burmese amber in scientific research (see Haug et al., 2020). The amber pieces used in this study were bought by private collectors 4–5 years ago. Therefore, the study of these amber pieces can be evaluated as unproblematic with respect to the current political situation in Myanmar."
Anisyutkin & Perkovsky (2022) *Cretaceous Research*	"All amber inclusions described in this paper were acquired legally in the Kachin State (Myanmar) by [name redacted] from local collectors, miners, or in the mineral shop in 2012 and 2014."
Baranov *et al.* (2022) *Bulletin of Geosciences*	"...specimens were legally acquired via the platform eBay by [name redacted] from the trader burmite-miner (see part of the discussion on amber trade from Myanmar e.g. Haug et al. 2020)."
Tihelka *et al.* (2022) *Insect Systematics and Diversity*	"The amber pieces were purchased in late 2016 from a Myanmar amber dealer whose family has been working in the amber business for many years. The material was mined prior to the start of the armed conflict in the Hukawng Valley, and thus the material is open to legitimate study, with respect to the laws of Myanmar and China (Engel 2020)"

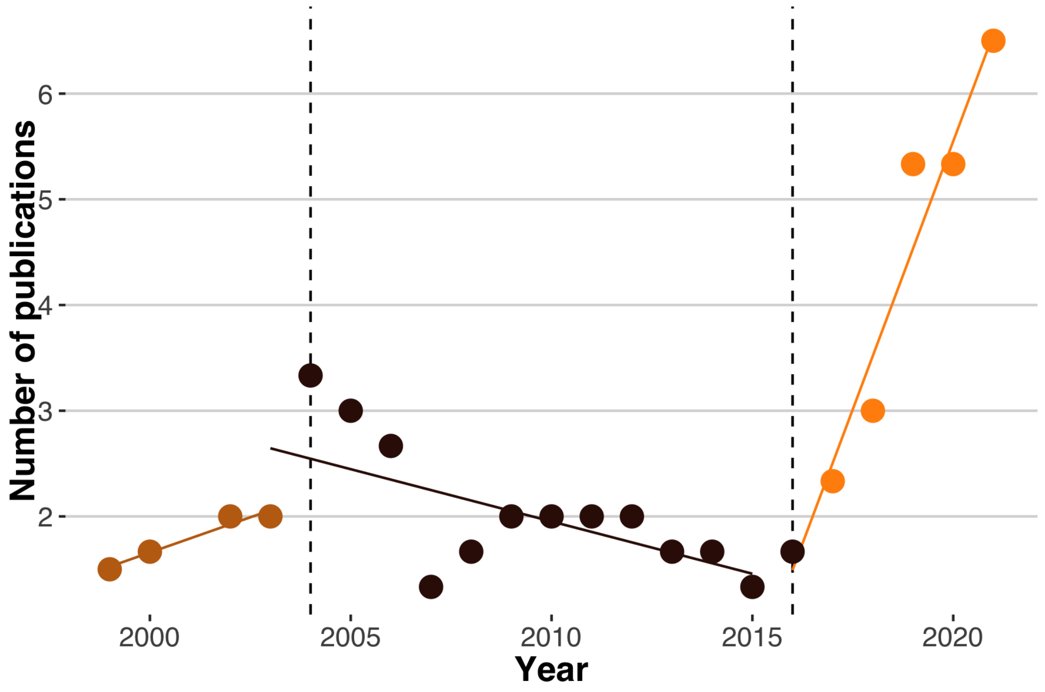

Fig S1. Trends in Myanmar non-amber publication activity from 1999-2021. Number of publications is measured by a 3-year rolling average to account for the peer review process. Breakpoints in the time series (at 2004 and 2016) are represented by vertical dotted lines.

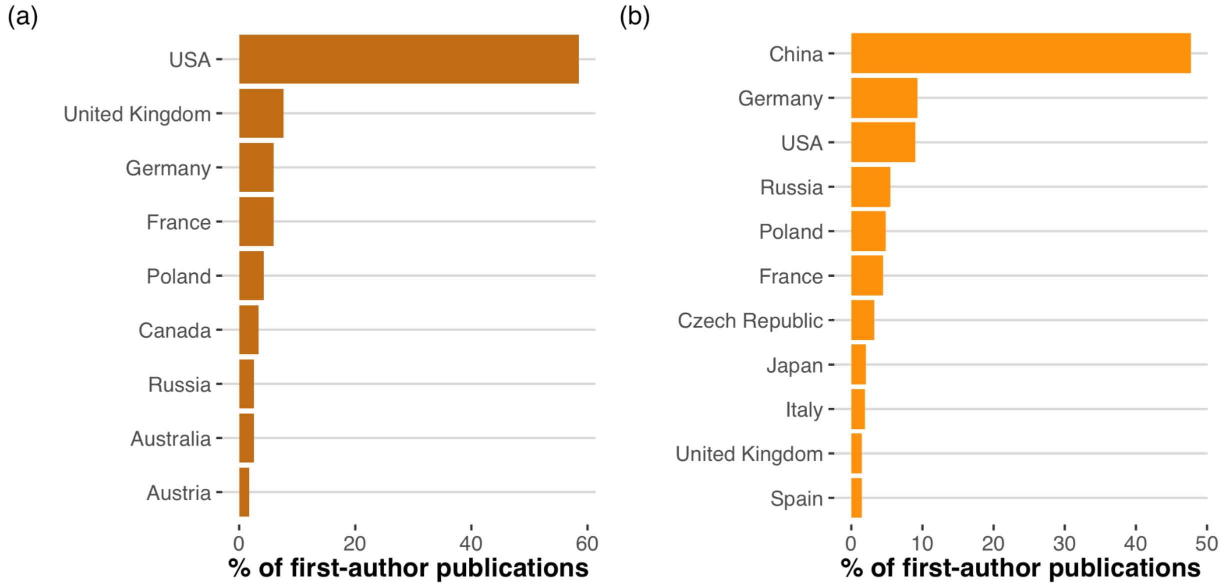

Fig S2. Percentage of first author publications per country on papers describing Myanmar amber research for (a) pre-2014 (n=*118*), (b) post-2014 (n=*874*).

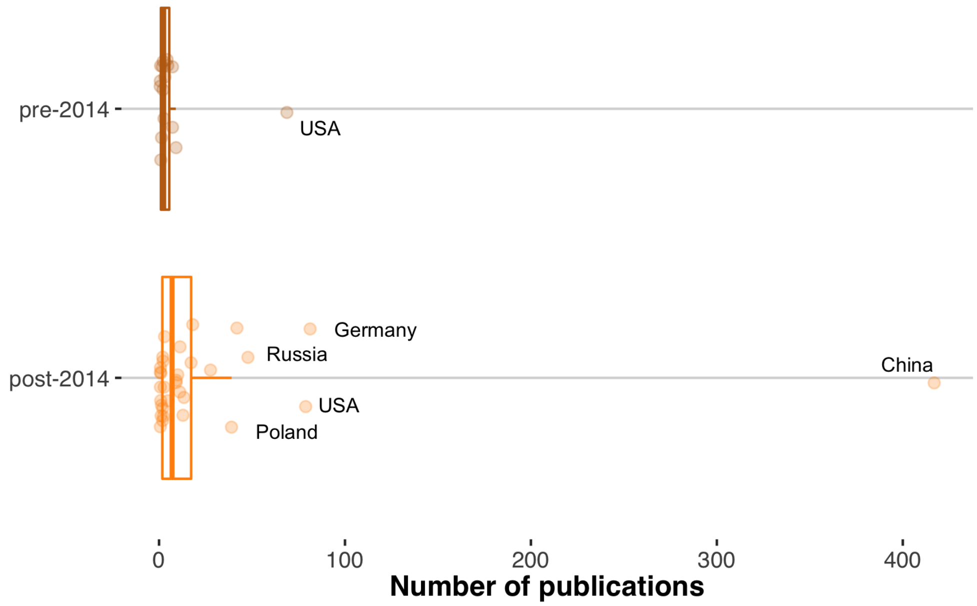

Fig S3. Distribution of number of publications per country before and after 2014. Each point represents a country that was listed as the affiliate country for the lead author for at least one publication. Countries that had significantly higher numbers of publications (outliers) are labeled.

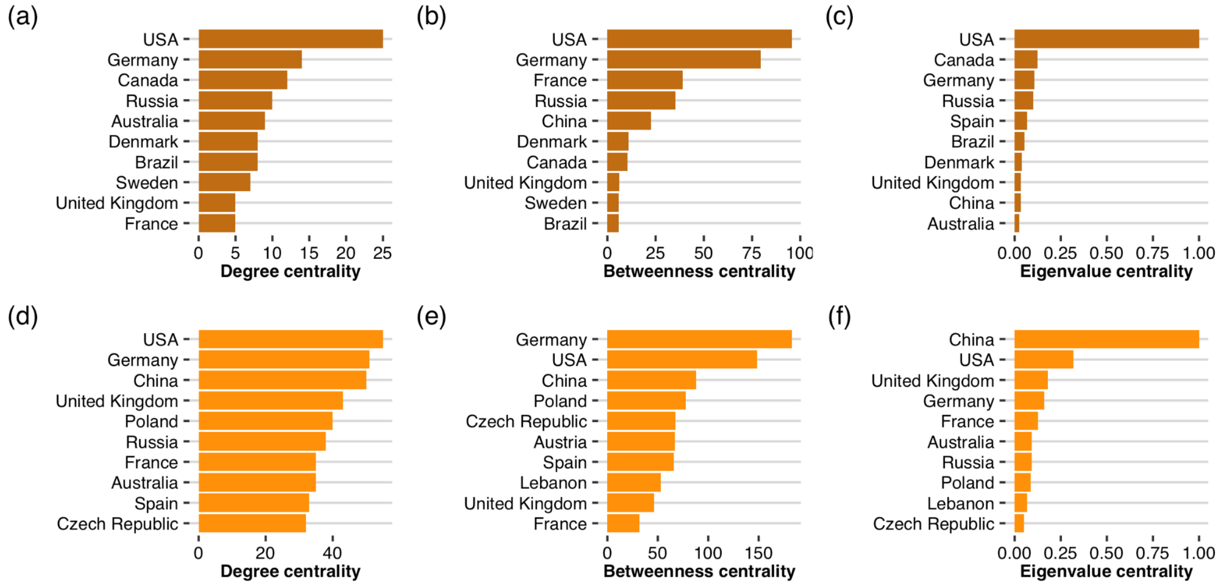

Fig. S4 Centrality measures for (a-c) pre-2014 and (d-f) post-2014. Only the top 10 countries for each measure are shown.

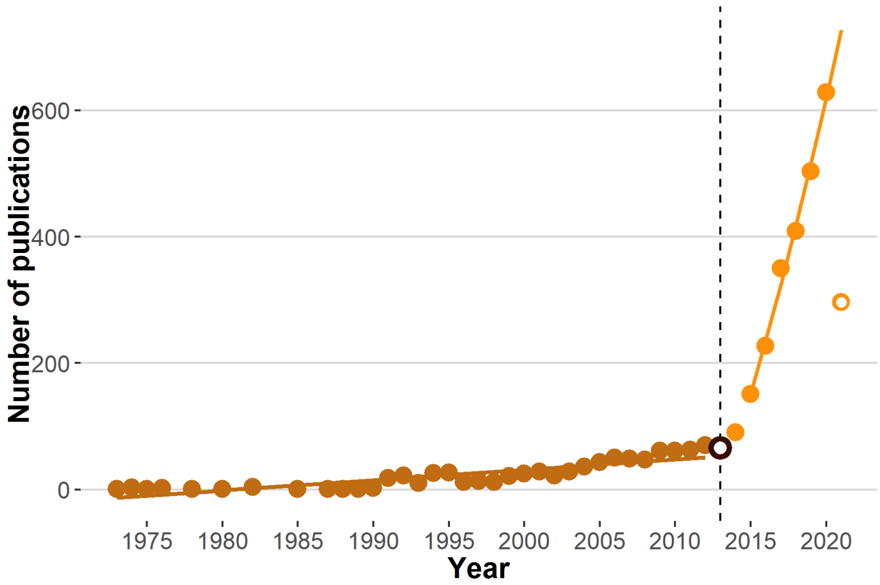

Fig. S5. Trends in publication activity by researchers based in Myanmar (1970-2020). Breakpoints in the time series are represented by the dotted line. Data obtained from the Web of Science.
